# Research trends and knowledge structure of ferroptosis in hearing loss: A bibliometric analysis (2012–2026)

**DOI:** 10.1097/MD.0000000000051252

**Published:** 2026-10-30

**Authors:** Feng Qiu, Ziyuan Chen, Huan Xie, Wusong Huang, Bin Ai

**Affiliations:** a Department of Otolaryngology Head and Neck Surgery, The First Affiliated Hospital of Fujian Medical University, Fuzhou, Fujian, China; b Department of Clinic Medicine, Nanchang Medical College, Nanchang, Jiangxi, China; c Department of Geriatric Medicine, The Third Affiliated People’s Hospital of Fujian University of Traditional Chinese Medicine, Fuzhou, Fujian, China; d ENT Department, The Third Affiliated People’s Hospital of Fujian University of Traditional Chinese Medicine, Fuzhou, Fujian, China.

**Keywords:** bibliometric analysis, cochlear injury, ferroptosis, GPX4, hearing loss, lipid peroxidation, oxidative stress

## Abstract

**Background:**

Hearing loss affects over 1.5 billion people worldwide and is a major public health issue. Ferroptosis, an iron-dependent cell death pathway, has been increasingly implicated in cochlear injury. However, the research landscape and evolving trends in this field have not been systematically characterized.

**Methods:**

Publications published between 2012 and 2026 were retrieved from the Web of Science Core Collection. Bibliometric and visualized analyses were performed using CiteSpace, VOSviewer, and the Bibliometrix R package.

**Results:**

A total of 75 publications (published up to March 30, 2026) were included in the final analysis. The current literature exhibits a substantial geographical concentration, with China contributing 85.3% of the total output, while international collaboration remains limited. This indicates a lack of geographically balanced global evidence in the current field. Current research mainly focuses on cisplatin-induced ototoxicity and noise-induced hearing loss; age-related hearing loss, hereditary hearing loss, and sudden sensorineural hearing loss have received comparatively less attention. Keyword burst analysis shows that research interests have gradually shifted from general oxidative stress mechanisms to specific molecular targets, including glutathione peroxidase 4, with growing attention to clinical and translational applications. Existing studies suggest that ferroptosis may contribute to inner ear injury through 3 interconnected processes: impaired antioxidant defense systems, dysregulated iron metabolism, and enhanced lipid peroxidation. These processes also interact with other regulated cell death pathways such as apoptosis, autophagy, and inflammation.

**Conclusion:**

This bibliometric analysis provides an overview of the current research on ferroptosis in hearing loss. However, the findings reveal a heavily geographically concentrated research output, emphasizing the need for broader international collaboration to establish more universally applicable evidence. The findings identify emerging research directions and may offer useful references for future studies on ferroptosis-related mechanisms and therapeutic strategies in otological disorders.

## 1. Introduction

According to the 2021 World Health Organization World Report on Hearing, more than 1.5 billion people worldwide are affected by hearing loss, and approximately 430 million have moderate to severe hearing impairment.^[1]^ As the population ages, environmental noise exposure increases, and ototoxic drugs are more widely used, hearing loss has become a major global public health concern.^[2]^ Common types of hearing loss include noise-induced hearing loss, age-related hearing loss, drug-induced hearing loss, hereditary hearing loss, and sudden sensorineural hearing loss.^[3,4]^ Studies have confirmed that irreversible damage to cochlear hair cells and spiral ganglion neurons is the core pathological change shared by all these types of hearing loss, but the complex molecular mechanisms behind this damage are not fully understood.^[5,6]^

Oxidative stress is considered one of the key mechanisms of cochlear injury.^[7–9]^ Ferroptosis is a newly identified form of regulated cell death, characterized by iron-dependent lipid peroxidation and accumulation of lipid reactive oxygen species.^[10–13]^ Since Dixon et al first proposed the concept in 2012, ferroptosis has been widely studied in cancer biology, neurodegenerative diseases, ischemia-reperfusion injury, and other fields.^[12,14]^ In recent years, more and more studies have suggested that ferroptosis plays an important role in hearing loss. However, current research in this field is still limited and scattered, and there is a lack of systematic global analysis of its research trends and development patterns.^[15]^

Therefore, based on data from the Web of Science Core Collection (WoSCC), we conducted a bibliometric analysis to summarize the global research progress of ferroptosis in hearing loss. We analyzed publication trends, international cooperation patterns, research hotspots, etiological distribution, and potential molecular mechanisms. This study is intended to provide a comprehensive understanding of the current research status and to offer insights for future studies on ferroptosis-related mechanisms, therapeutic strategies, and clinical applications in hearing disorders.

## 2. Materials and methods

### 2.1. Data source and search strategy

We retrieved literature from the WoSCC on April 10, 2026, covering the period from January 1, 2012 to March 30, 2026. The approximately 10-day gap between the end of the inclusion period and the search date was intentionally incorporated to account for routine database indexing delays, ensuring that all relevant publications up to March 2026 were fully captured.

The search strategy was constructed as follows: TS = (“ferroptosis” OR “ferroptotic”) AND TS = (“hearing loss” OR “deafness” OR “sensorineural hearing loss” OR “ototoxicity” OR “cochlea injury” OR “hair cell death” OR “auditory injury”). These search terms were developed based on a combination of medical subject headings concepts related to auditory disorders and key terminology frequently observed in recent seminal papers. Prior to the formal search, pilot searches were conducted to test alternative terminology and spelling variants, confirming that the current combination of terms maximized retrieval sensitivity for studies specifically targeting ferroptosis-mediated inner ear damage.

The database search was strictly limited to the science citation index expanded (SCI-E) index within the WoSCC. This SCI-E restriction was implemented directly in the database search settings prior to exporting the results, rather than being applied during the manual literature screening phase. Furthermore, document types were limited to Article and Review, and the language was restricted to English.

### 2.2. Literature screening

Duplicate records were removed using EndNote software. Two researchers independently screened the retrieved studies according to the Preferred Reporting Items for Systematic Reviews and Meta-Analyses (PRISMA) guidelines.^[16]^ It is important to clarify that while we employed the PRISMA framework specifically to guide our systematic literature screening and selection process to ensure transparency and reproducibility, this study is fundamentally a bibliometric analysis rather than a conventional systematic review aimed at systematic evidence synthesis.

Two researchers independently screened the retrieved studies sequentially: first by title and abstract, followed by a full-text assessment for eligibility. Any disagreement during the screening process was discussed and resolved by a third researcher. The inclusion criteria were as follows: studies directly investigating the relationship between ferroptosis and hearing loss or auditory system injury, including cochlear hair cells and spiral ganglion neurons; publication types classified as original research articles or reviews; studies indexed in SCI-E; and articles published in English.

The exclusion criteria included: duplicate records; non-research publications, such as editorials, corrections, conference abstracts, letters, and comments; and studies that only mentioned ferroptosis or hearing loss without examining their potential association.

### 2.3. Bibliometric analysis

CiteSpace version 6.4.R1 (Drexel University, USA),^[17]^ VOSviewer version 1.6.20 (Leiden University, Netherlands),^[18]^ and Bibliometrix R version 4.1.0 (University of Naples Federico II, Italy)^[19]^ were used for analysis.

Prior to network extraction and clustering, a rigorous keyword normalization procedure was implemented. A custom thesaurus file was applied to manually merge obvious synonyms, spelling variants, and hyphenation differences (e.g., standardizing “hearing-loss” to “hearing loss,” and “hair-cells” to “hair cells”).

CiteSpace was used for co-citation analysis, clustering, keyword burst detection, and betweenness centrality calculation. To ensure full reproducibility, the specific parameters in CiteSpace were set as follows: the timespan was set from 2020 to 2026 (since no relevant articles were published before 2020), with a slice length of 1 year. Node types were selected individually based on the specific analysis (including Author, Institution, Country, Keyword, and Cited Reference). The complete selection criteria were configured as: g-index (k = 25), link retaining factor = 2.5, links per node = 10, look back years = 5, and e = 1.0. For network pruning, the Pathfinder algorithm was applied to minimize excessive incidental links and highlight the core structural features of the networks.

VOSviewer was used to visualize collaboration networks of countries, institutions, authors, and keywords. The full counting method was adopted, and association strength was used for standardization. The minimum number of publications was set to 1 for country, institution, and author networks, and the minimum occurrence frequency was set to 3 for the keyword co-occurrence network.

The Bibliometrix R package was used to analyze annual publication volume, high-yield authors, institutions/countries.

### 2.4. Network metrics

The standardized algorithms implemented in CiteSpace were used to evaluate the importance of nodes within collaboration networks and co-citation networks.

Nodes with a betweenness centrality value > 0.1 were considered important nodes in the network. The reliability of clustering results was evaluated by modularity (*Q* value) and silhouette coefficient (*S* value). Generally, *Q* > 0.3 and *S* > 0.7 indicate meaningful and reliable clustering structures. In this study, the actual *Q* and mean silhouette (*S*) values for the specific generated clusters are explicitly reported in the corresponding Results sections to confirm structural reliability.

### 2.5. Etiological distribution analysis

Two researchers independently classified all included studies according to the etiology of hearing loss. This classification was performed strictly at the publication level rather than the experiment or model level. The etiological categories were defined as mutually exclusive. For the minority of publications covering more than 1 hearing loss etiology, the classification was assigned based on the predominant disease model that the study’s central hypothesis aimed to address.

The operational definitions for each category were established as follows: drug-induced: hearing loss primarily resulting from exposure to ototoxic pharmacological agents (e.g., cisplatin, aminoglycosides); noise-induced: auditory injury caused by acute or chronic exposure to high-intensity sound; age-related: progressive hearing impairment associated with the natural aging process; hereditary: hearing dysfunction driven by specific genetic mutations or inherited traits; and sudden sensorineural hearing loss: rapid onset of unexplained hearing reduction typically occurring within 72 hours.

Disagreements were reviewed and resolved through discussion with a third researcher. Inter-rater agreement was evaluated using Cohen kappa coefficient. The obtained kappa value of 0.86 indicated excellent agreement between the 2 researchers. The distribution of different etiological categories was visualized with a circular chart.

### 2.6. Data availability statement and ethical considerations

The data supporting this study, including literature records, network files, and bibliometric matrices, were all obtained from the WoSCC using the search strategy described above. The generated analysis files and visualization data are available from the corresponding author upon reasonable request. Since this study was based on published literature and did not involve human subjects, animal experiments, or identifiable personal information, ethical approval was not required.

## 3. Results

### 3.1. Literature screening and annual publication trends

The literature screening process based on the PRISMA guidelines is shown in Figure 1. Specifically, a total of 76 records were initially retrieved. No duplicate records were identified. During the title and abstract screening phase, 1 record was excluded because it was a correction document (classified as a non-research publication). Consequently, 75 full-text articles were sought and assessed for eligibility. All 75 articles met the inclusion criteria during the full-text screening and were finally included in the bibliometric analysis. Among these 75 publications, 59 were original research articles and 16 were reviews.

**Figure 1.**
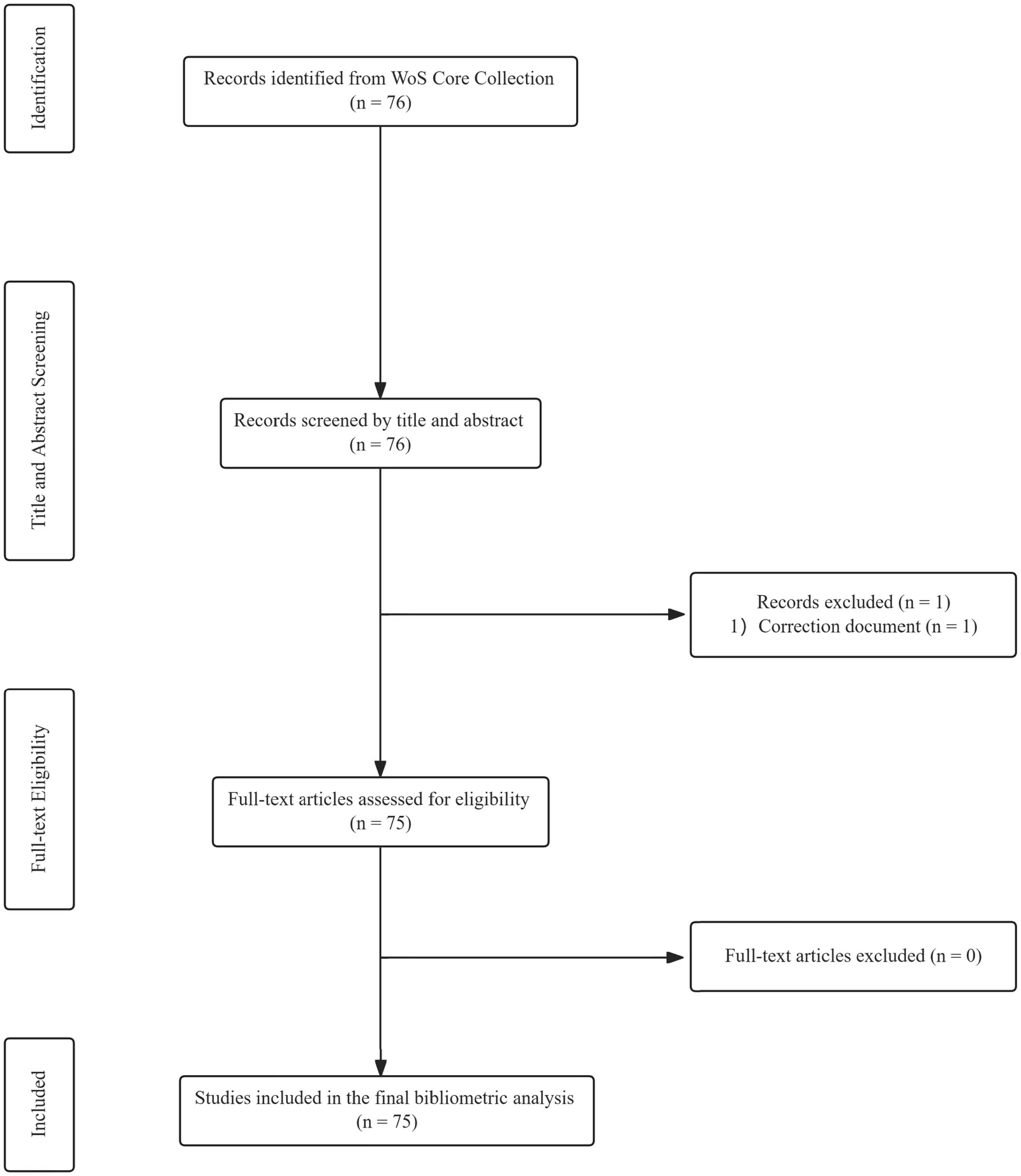
Flowchart of literature retrieval and screening process. The literature retrieval and screening workflow for studies related to ferroptosis and hearing loss is based on the WoSCC database. The diagram illustrates the processes of identification, screening, eligibility assessment, and final inclusion of publications according to predefined criteria. n = number of records, WoSCC = Web of Science Core Collection.

No relevant literature was published between 2012 and 2019. The first article was published in 2020, followed by rapid growth after 2022, reaching a peak of 23 articles in 2024, 16 articles in 2025, and 10 articles already included in 2026 (representing a partial year through March 30). This indicates that ferroptosis and hearing loss is a rapidly emerging frontier research field in the past 5 years (Fig. 2).

**Figure 2.**
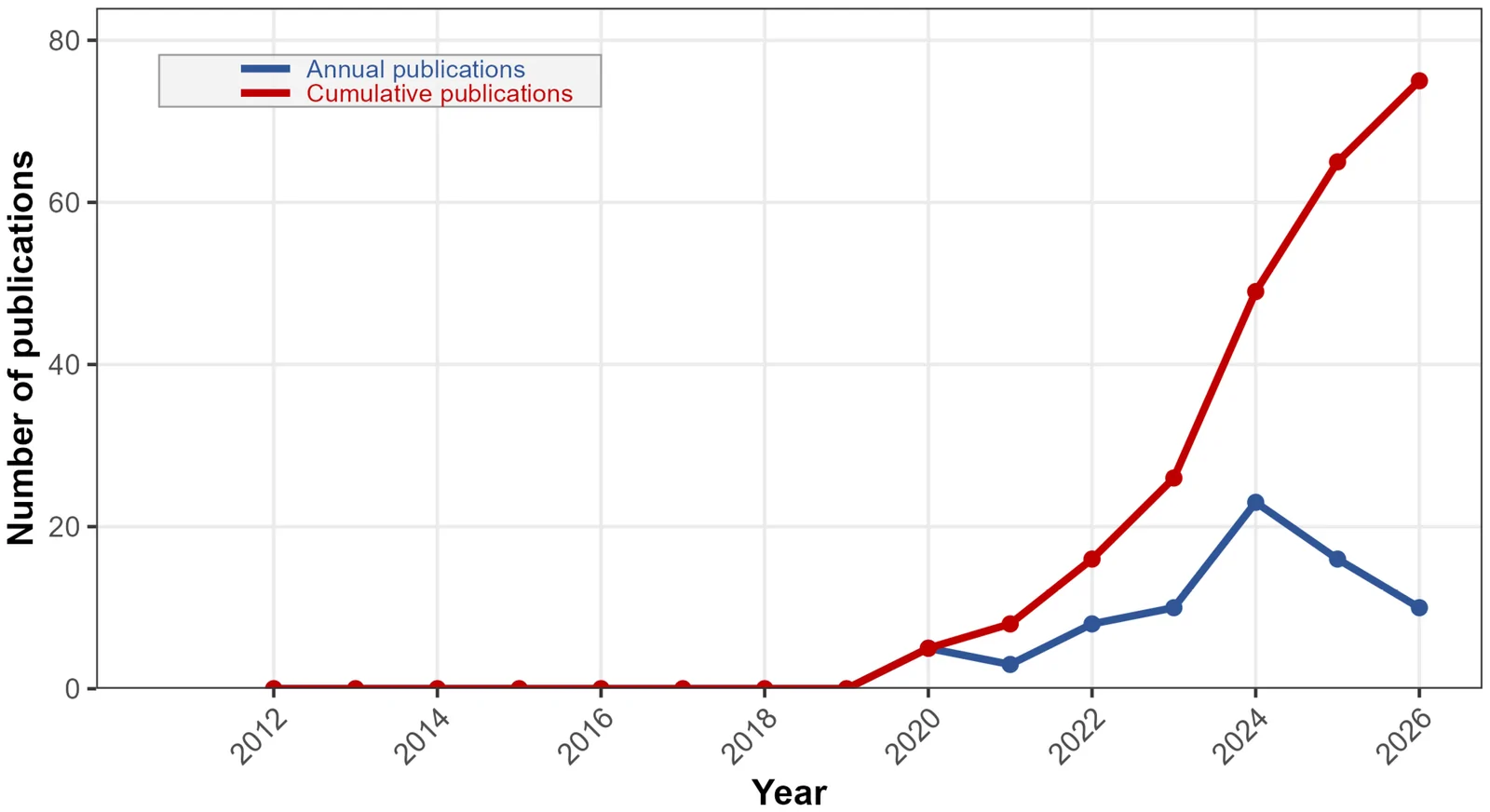
Annual publication trends in ferroptosis-related hearing loss research from 2012 to 2026. The blue line represents the annual number of publications, whereas the red line indicates the cumulative number of publications.

### 3.2. Country/region collaboration network

The study involves 17 countries or regions. China ranks first globally with 64 publications (85.3%), followed by the United States with 6 publications (8.0%) and South Korea with 3 publications (4.0%). International collaborations primarily occur among China–United States, United States–South Korea, and China–South Korea, but the overall level of international cooperation remains extremely limited. A global academic collaboration system has not yet been established. The collaboration network map of major publishing countries is shown in Figure 3.

**Figure 3.**
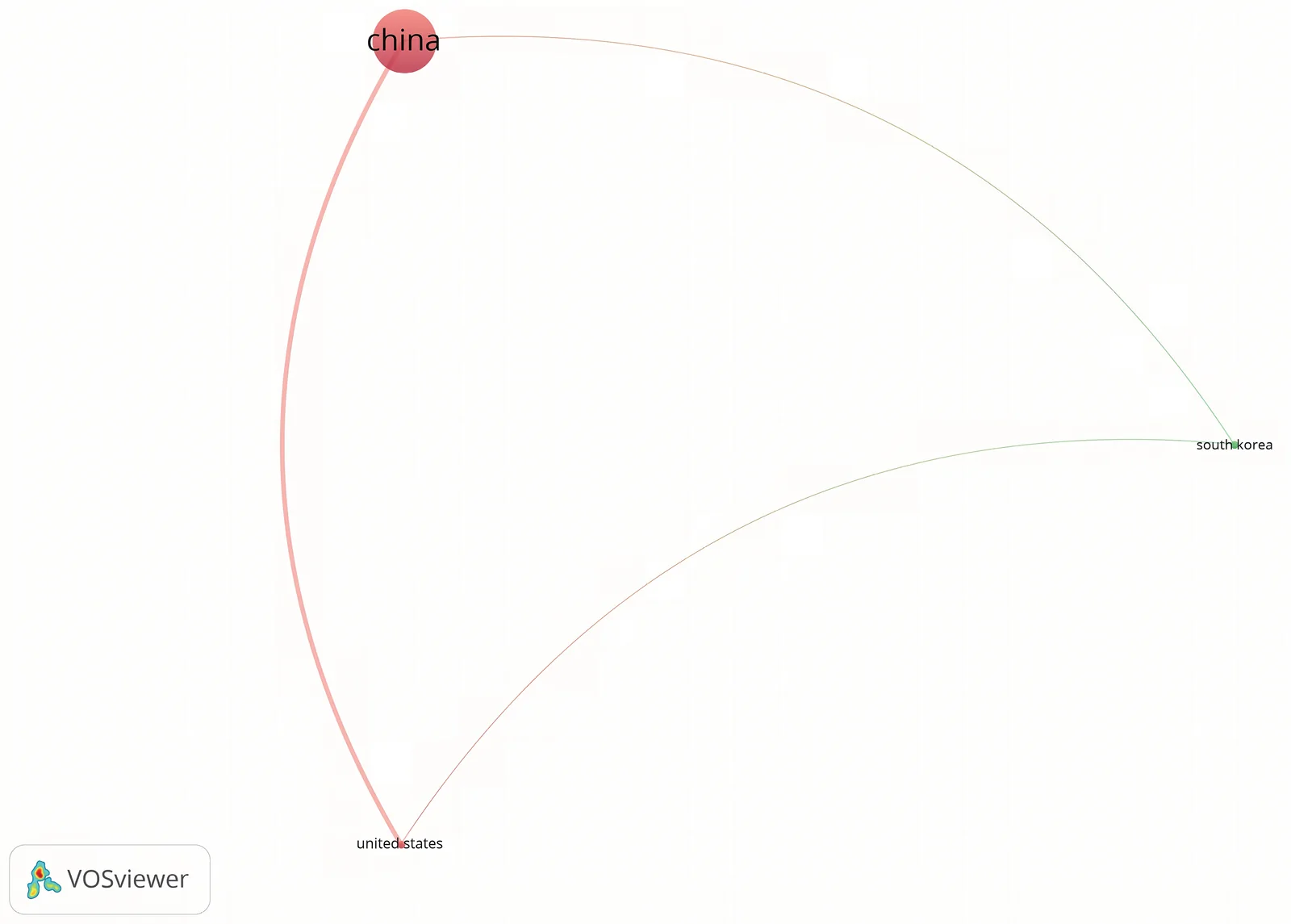
Global distribution and country collaboration network of ferroptosis-related hearing loss research. Geographic distribution of publications by country/region. Darker colors indicate higher publication output. International collaboration network among countries. Node size represents publication quantity, while link thickness indicates collaboration strength between countries.

### 3.3. Analysis of institutions and authors

The top 10 research institutions are all from China. Fudan University and Shandong University tie for first place with 9 publications each; Fudan University has the highest total citation frequency (207 times); Shandong University has the highest total collaboration strength (26), indicating its core position in the institutional collaboration network. The publication volume, citation frequency, and collaboration strength of the top 10 institutions are shown in Table 1.

**Table 1:** Top 10 institutions contributing to ferroptosis research in hearing loss.

Rank	Institution	Documents	Citations	Total link strength
1	Fudan University	9	207	4
2	Shandong University	9	140	26
3	Shandong First Med University	7	112	18
4	Shenzhen University	5	111	2
5	Southeast University	5	101	21
6	Nantong University	4	101	20
7	Air Force Med University	3	47	0
8	Beijing Inst Technol	3	35	13
9	Binzhou Med University	3	35	4
10	Case Western Reserve University	3	65	4

High-yield authors: Li Wen and Nie Guohui each have 5 publications. Li Wen has the highest influence with 228 citations. Co-cited authors: Dixon SJ (53 times), the proposer of the ferroptosis concept, ranks first; the top 10 high-yield authors and co-cited authors are shown in Table 2.

**Table 2:** Top 10 productive authors and co-cited authors.

Rank	Author	Documents	Citations	Coauthor	Citations
1	Li, Wen	5	228	Dixon, Sj	53
2	Nie, Guohui	5	111	Chen, X	30
3	He, Yingzi	4	177	Mei, Hl	30
4	Chai, Renjie	4	101	Stockwell, Br	26
5	Gao, Wei	4	69	Zheng, Zw	25
6	Yuan, Hao	4	69	He, Zh	23
7	Zhao, Liping	3	177	Yang, Ws	23
8	Zheng, Zhiwei	3	167	Hu, B	21
9	Zheng, Qingyin	3	65	Jian, Bq	20
10	Fu, Xiaolong	3	54	Rybak, Lp	20

### 3.4. Analysis of institutional and author collaboration networks

Institutional collaboration analysis shows that research on ferroptosis-related hearing loss is mainly contributed by Chinese institutions, with Fudan University and Shandong University being the most productive. However, collaboration between institutions from different countries or regions is still limited, meaning international cooperation networks have not been fully developed.

The author collaboration network presents several relatively independent research groups. Cooperation within each team is close, but connections between different research groups are weak. Co-citation analysis shows that Dixon SJ is the most frequently cited author, which reflects his foundational contribution to the ferroptosis concept. In addition, auditory pathology researchers such as Rybak LP and Hu B also have high co-citation frequencies, indicating the gradual integration of ferroptosis research and auditory injury research (Fig. 4).

**Figure 4.**
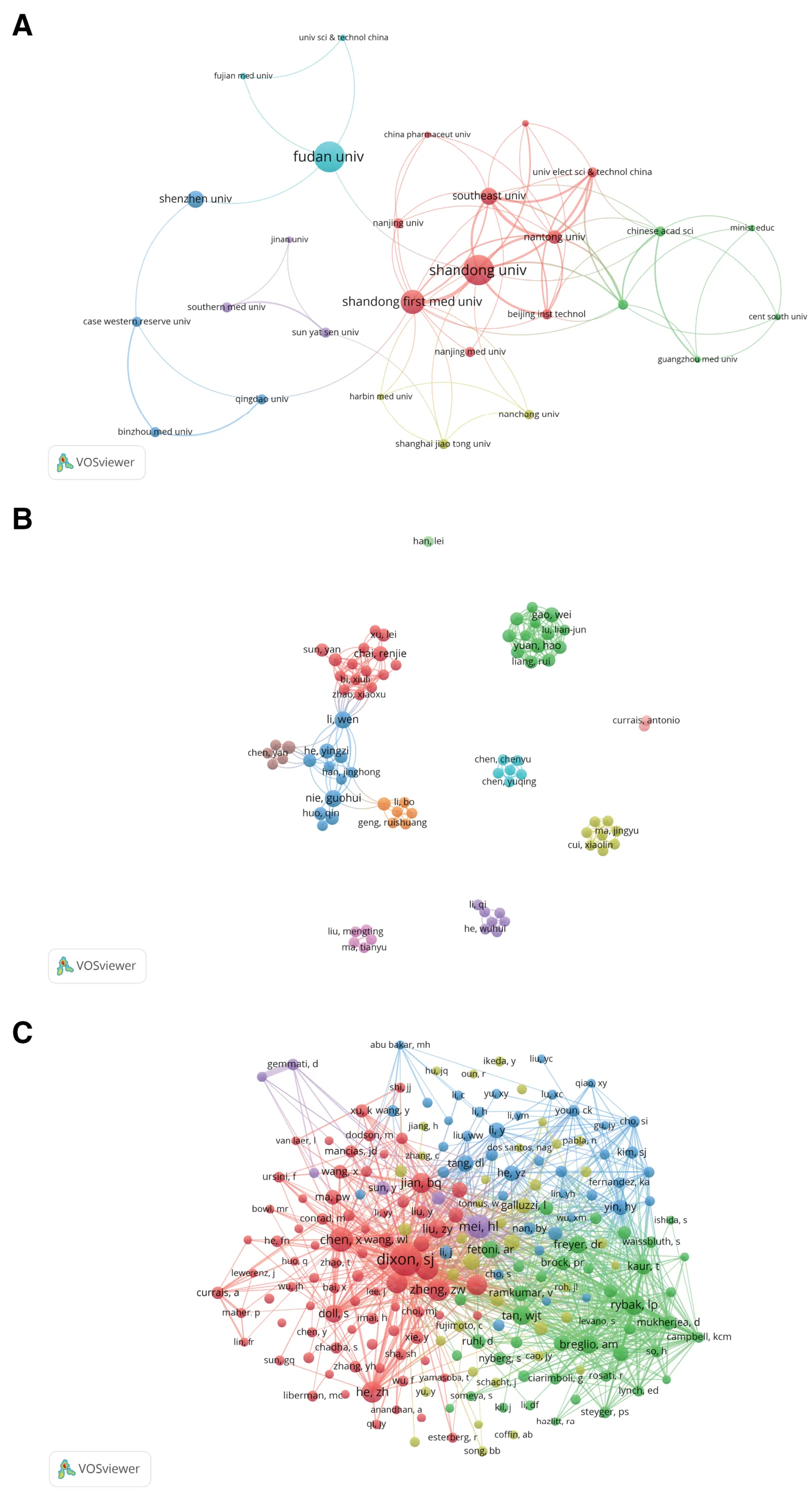
Institutional and author collaboration analysis. (A) Institutional collaboration network of ferroptosis-related hearing loss research. Node size represents publication output, while connecting lines indicate collaborative relationships among institutions. (B) Author collaboration network. Different colors represent distinct research clusters and collaborative groups. (C) Co-cited author network. Node size reflects co-citation frequency, indicating the academic influence of individual authors within the field.

### 3.5. Co-cited reference analysis

Co-citation analysis identified several key references in this field. The 2012 Cell study by Dixon et al is the most frequently co-cited reference, which is consistent with its role as the foundational paper on ferroptosis. Other highly cited studies include those by Mei HL (2020, J Cell), Jiang X (2021, Mol Cell), Liu ZY (2024, Mol Ther) and Hu B (2020, Biochem), which mainly focused on the relationship between ferroptosis and auditory injury (Fig. 5A).

**Figure 5.**
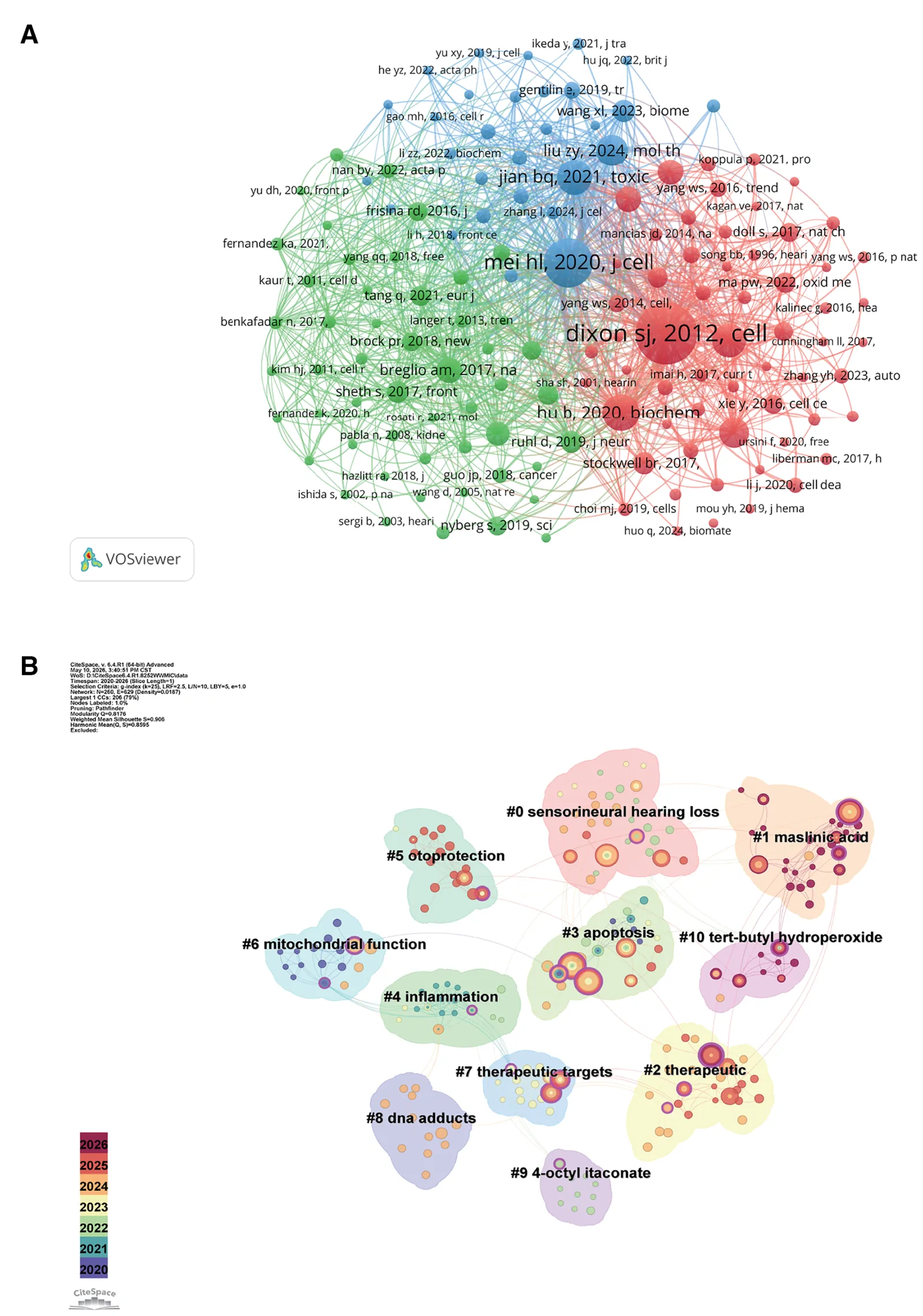
Co-cited reference analysis. (A) Co-citation network of references in ferroptosis-related hearing loss research. Node size represents co-citation frequency, while link thickness indicates co-citation strength between references. (B) Reference clustering analysis identifying major research themes. Different colors represent distinct thematic clusters associated with ferroptosis mechanisms, oxidative stress, cochlear injury, and therapeutic interventions.

Clustering analysis identified 11 main research clusters with excellent structural reliability (Figure 5B, Modularity *Q* = 0.8176, Weighted Mean Silhouette *S* = 0.906). Among them, Cluster #0 (“sensorineural hearing loss”) is the largest cluster, mainly focusing on ferroptosis-related auditory injury. Cluster #1 (“maslinic acid”) is related to natural compounds and their protective effects. Cluster #2 (“therapeutic”) covers intervention studies targeting ferroptosis. Cluster #3 (“apoptosis”) includes studies on the relationship between ferroptosis and other cell death pathways.

In addition, Cluster #4 (“inflammation”) and Cluster #6 (“mitochondrial function”) reflect growing research interest in inflammatory responses and cellular stress processes. Newly emerged clusters such as Cluster #7 (“therapeutic targets”), Cluster #8 (“DNA adducts”), Cluster #9 (“4-octyl itaconate”) and Cluster #10 (“tert-butyl hydroperoxide”) represent the latest research directions. These findings indicate that recent studies have gradually expanded from general ferroptosis injury mechanisms to specific regulatory molecules and intervention approaches.

### 3.6. Keyword co-occurrence and cluster analysis

Table 3 lists the top 15 high-frequency keywords. The keyword co-occurrence network has a relatively close structure, and high-frequency keywords are mainly related to oxidative stress, ototoxicity, and cell death (Fig. 6A).

**Table 3:** Top 15 occurrences keywords.

Rank	Keyword	Occurrences	Total link strength
1	ferroptosis	51	223
2	oxidative stress	25	112
3	ototoxicity	23	116
4	cisplatin	22	127
5	hearing loss	20	89
6	mechanisms	17	75
7	hair cell	16	66
8	apoptosis	13	72
9	death	11	58
10	cancer	10	47
11	inflammation	10	54
12	autophagy	9	49
13	lipid peroxidation	9	43
14	expression	8	31
15	induced ototoxicity	8	36

**Figure 6.**
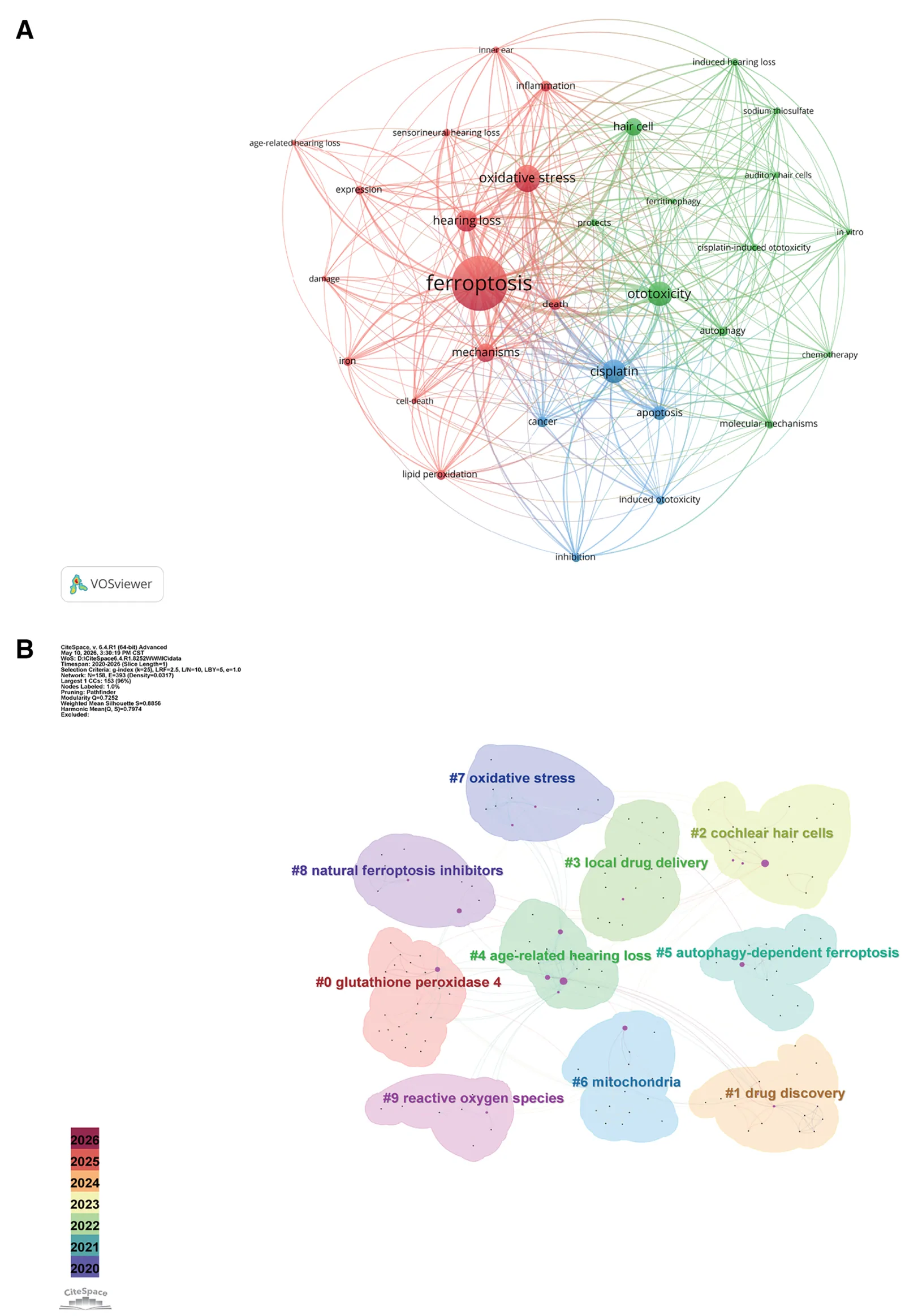
Keyword co-occurrence and clustering analysis of ferroptosis-related hearing loss research. (A) Keyword co-occurrence network. Node size represents keyword frequency, while link thickness indicates co-occurrence strength. Different colors represent distinct thematic clusters. (B) Keyword clustering analysis of ferroptosis-related hearing loss research. Different colors represent distinct thematic clusters identified through keyword clustering analysis. Major clusters included “drug discovery,” “mitochondria,” “reactive oxygen species,” “glutathione peroxidase 4,” “natural ferroptosis inhibitors,” “oxidative stress,” “cochlear hair cells,” “local drug delivery,” “autophagy-dependent ferroptosis,” and “age-related hearing loss.” The clustering results illustrate the major thematic structure and evolving research directions within the field.

Thirty-two high-frequency keywords were included for cluster analysis, and 10 relatively stable clusters were generated (Modularity *Q* = 0.7252, Weighted Mean Silhouette *S* = 0.8856). Cluster #2 (“cochlear hair cells”), Cluster #4 (“age-related hearing loss”) and Cluster #7 (“oxidative stress”) are core research topics that have received continuous attention. Cluster #5 (“autophagy-dependent ferroptosis”) and Cluster #8 (“natural ferroptosis inhibitors”) are emerging research topics in recent years. Cluster #3 (“local drug delivery”) and Cluster #6 (“mitochondria”) reflect increasing attention to therapeutic methods and organelle-related mechanisms.

Notably, Cluster #0 (“glutathione peroxidase 4 [GPX4]”) forms an independent cluster, which is strongly associated with studies on molecular regulation of ferroptosis (Fig. 6B).

### 3.7. Keyword burst analysis

Keyword burst detection was performed to identify emerging research topics and changes in research attention over time (Fig. 7). Early burst keywords (2020–2021), including “iron,” “inflammation,” “cell death,” and “activation,” mainly reflected the initial research focus on iron metabolism, oxidative stress, and cell death-related processes.

**Figure 7.**
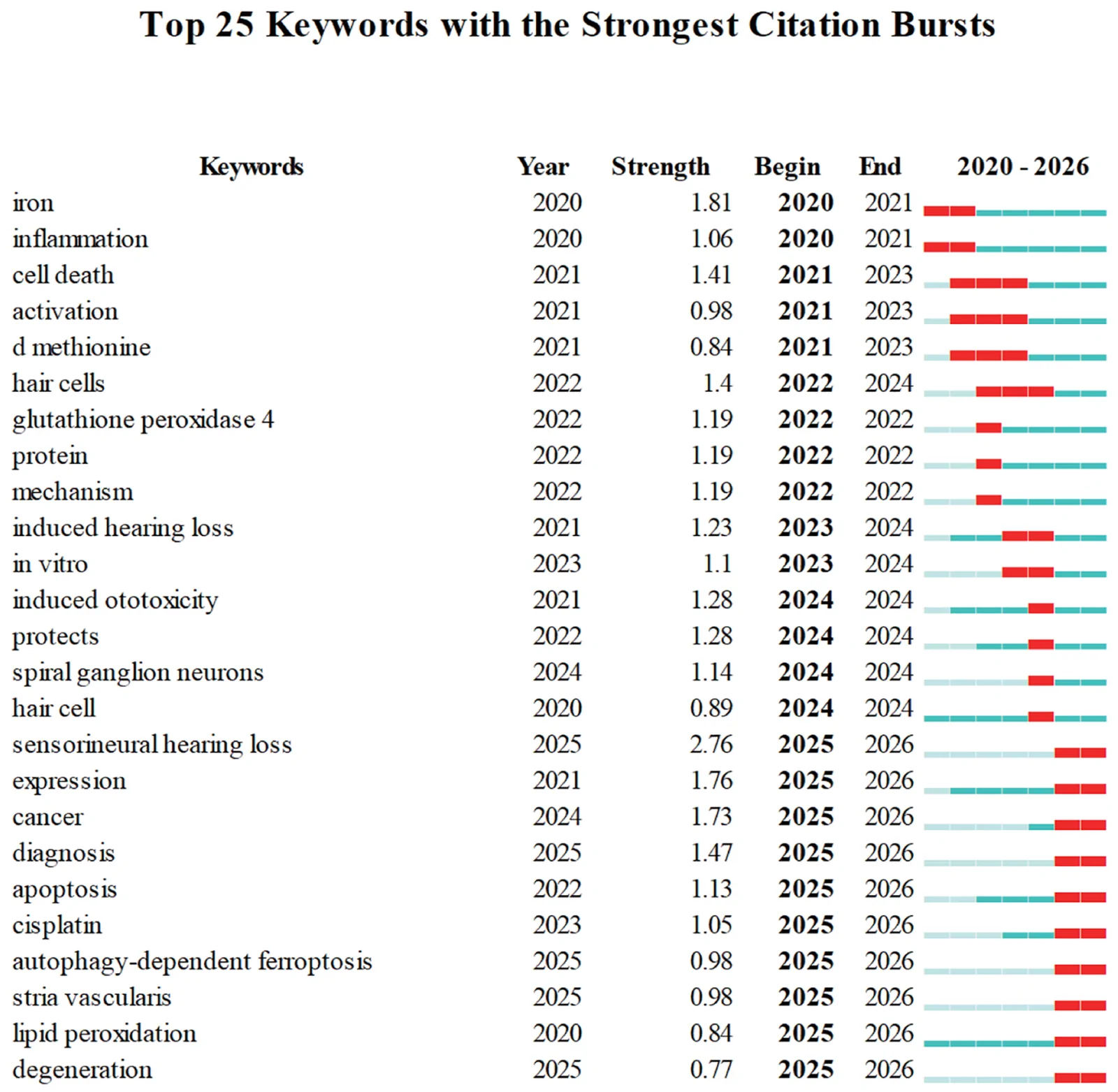
Top keywords with the strongest citation bursts. Visualization of keywords exhibiting the strongest citation bursts over time. Red bars indicate periods of intensive research attention, reflecting emerging hotspots and evolving thematic trends in ferroptosis-related hearing loss research.

Later, keywords such as “GPX4,” “hair cells,” “protein” and “in vitro” showed strong burst activity, indicating increasing attention to molecular regulation of ferroptosis and experimental studies of cochlear injury. These findings suggest that research interest has gradually shifted from general ferroptosis injury processes to specific molecular factors and cellular models.

### 3.8. Etiological distribution analysis

The distribution of different etiological types is shown in Figure 8. Drug-induced hearing loss accounts for the highest proportion (35/75, 46.67%), with cisplatin-induced ototoxicity being the most common subtype. Noise-induced hearing loss ranks second (21/75, 28.00%); age-related hearing loss accounts for 17.33% (13/75); hereditary hearing loss and sudden sensorineural hearing loss have relatively low proportions, at only 5.33% (4/75) and 2.67% (2/75), respectively.

**Figure 8.**
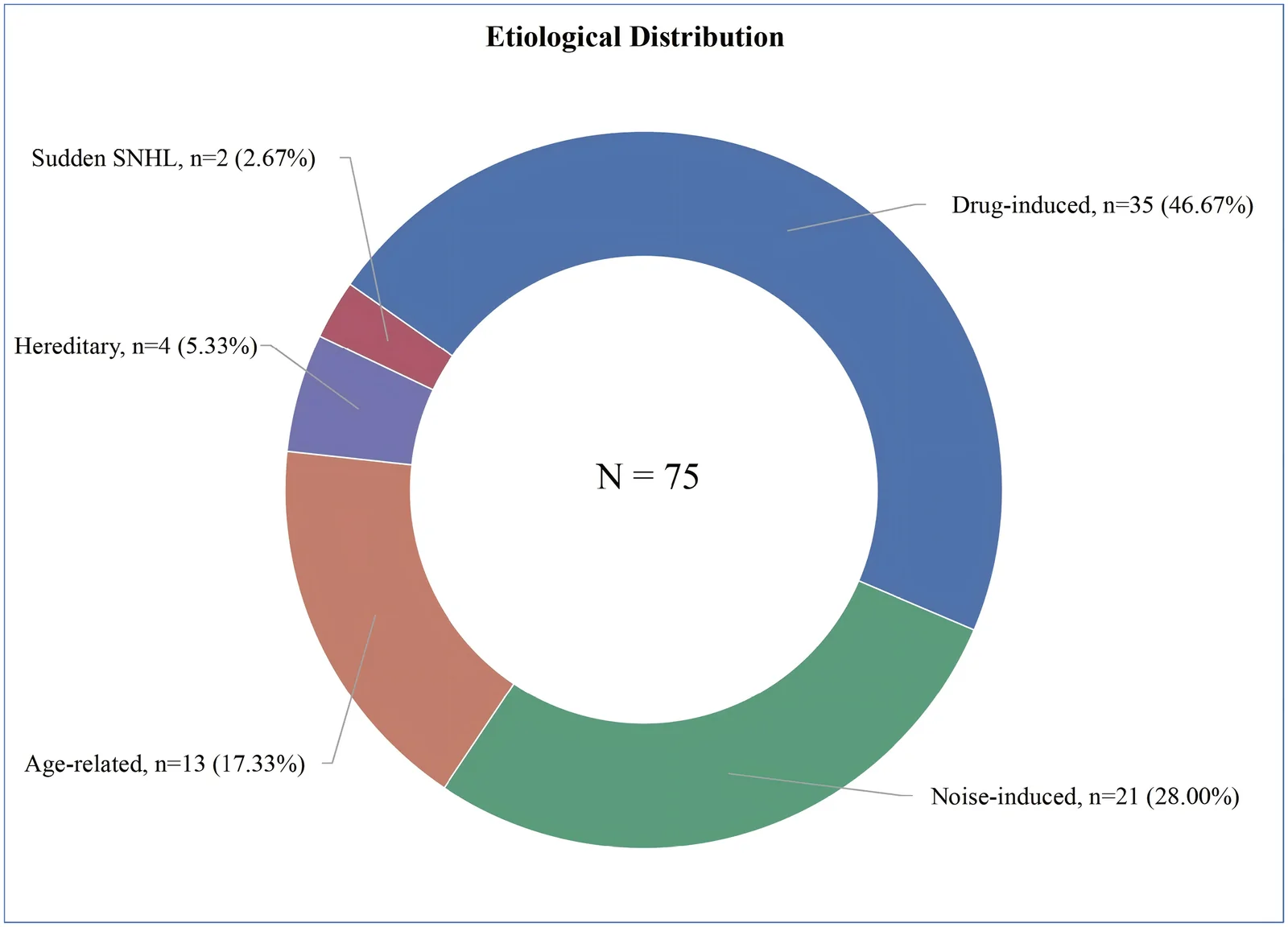
Etiological distribution of hearing loss studies associated with ferroptosis. Distribution of included studies according to hearing loss etiology, including cisplatin-induced ototoxicity, noise-induced hearing loss, age-related hearing loss, hereditary hearing loss, and sudden SNHL. SNHL = sensorineural hearing loss.

From the perspective of etiological proportion structure, existing research topics are highly concentrated on 2 exogenous injury models: drugs and noise, which together account for 74.67% (56/75) of all literature. In contrast, research on aging-related, hereditary, and sudden sensorineural hearing loss, which are clinically prevalent, is significantly insufficient in volume.

### 3.9. Mechanistic context of bibliometric hotspots

It should be noted that this section provides a contextual interpretation of the bibliometric results rather than a comprehensive systematic review. Through the analysis of highly cited publications (Section 3.5) and keyword clustering (Section 3.6) identified in our network, research on ferroptosis-related hearing loss mainly focuses on 3 biological processes: iron metabolism imbalance, impaired antioxidant defense, and dysregulated lipid peroxidation.

In terms of iron metabolism, previous studies have reported that increased expression of transferrin receptor (TFRC) and divalent metal transporter 1 (DMT1) promotes intracellular iron accumulation. In addition, nuclear receptor coactivator 4 (NCOA4)-mediated ferritinophagy enhances ferritin degradation and increases free iron release, while reduced ferroportin 1 (FPN1) expression decreases iron export. These changes together lead to iron overload and oxidative stress through iron-dependent reactions.

In terms of lipid metabolism, acyl-CoA synthetase long-chain family member 4 (ACSL4) is a widely studied ferroptosis regulator. Increased ACSL4 activity promotes the incorporation of polyunsaturated fatty acids into membrane phospholipids, which increases sensitivity to lipid peroxidation and ferroptosis-related cellular responses.

In addition, previous experimental studies have explored several ferroptosis regulation strategies, including ferrostatin-1 (Fer-1), iron chelators, and nuclear factor erythroid 2-related factor 2 (Nrf2)-related antioxidant approaches. These interventions have shown protective effects in experimental models of ferroptosis-related cell injury, suggesting potential directions for future inner ear protection research (Fig. 9).

**Figure 9.**
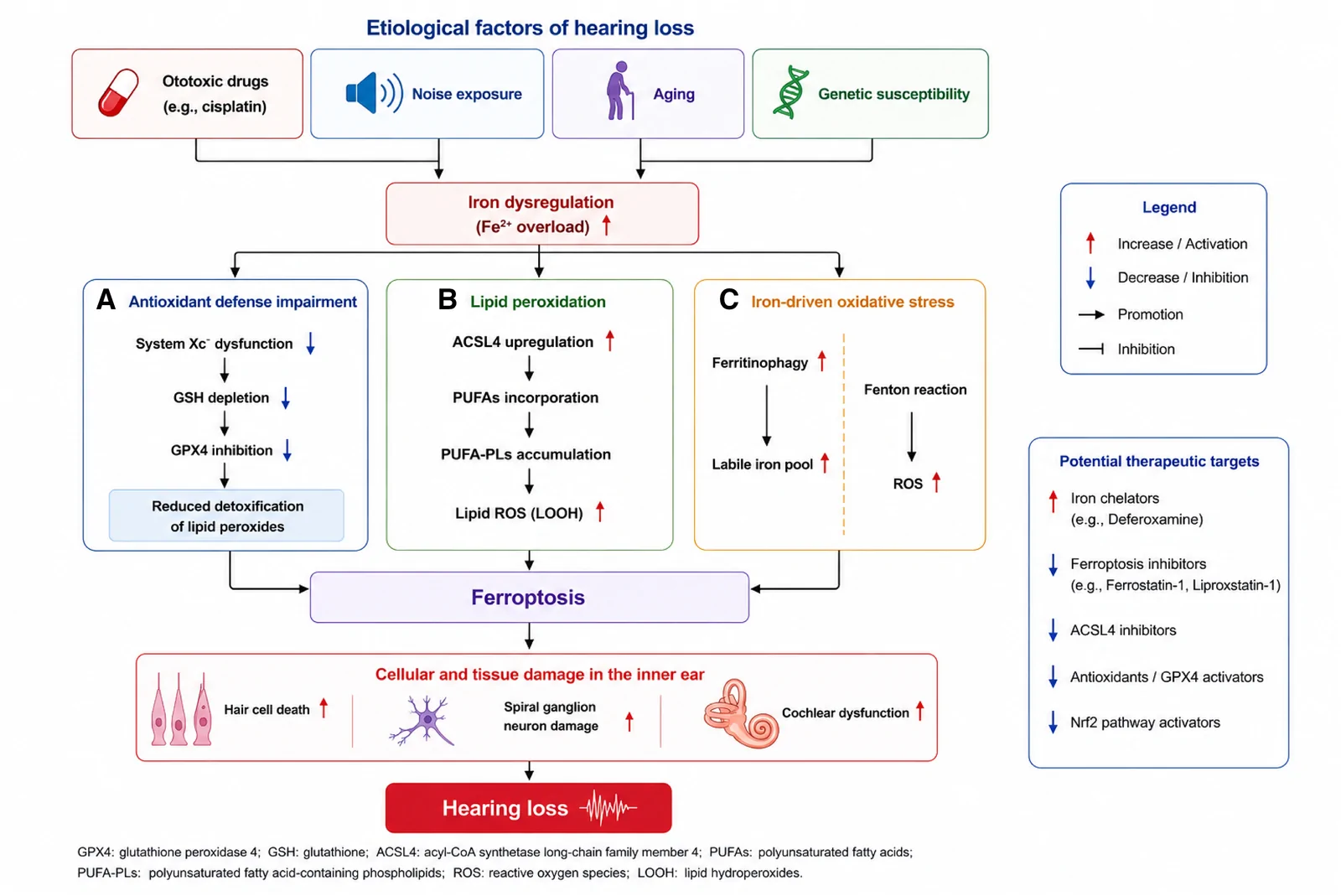
Mechanistic pathways of ferroptosis in hearing loss. Schematic illustration of the major molecular mechanisms underlying ferroptosis-mediated cochlear injury. Key pathways include iron metabolism dysregulation, glutathione depletion, GPX4 suppression, lipid peroxidation, mitochondrial dysfunction, oxidative stress accumulation, inflammatory activation, and cochlear hair cell death. ACSL4 = acyl-CoA synthetase long-chain family member 4, GPX4 = glutathione peroxidase 4, GSH = glutathione, LOOH = lipid hydroperoxide, PUFA = polyunsaturated fatty acids, PUFA-PL = polyunsaturated fatty acids-containing phospholipid, ROS = reactive oxygen species.

## 4. Discussion

### 4.1. A late-starting but rapidly developing field

Ferroptosis is an iron-dependent regulated cell death characterized by the accumulation of lipid peroxidation. Since it was first described by Dixon et al in 2012, it has received extensive attention in cancer biology, neurodegenerative diseases, ischemia-reperfusion injury, and other fields.^[12,13,20,21]^ However, research on the relationship between ferroptosis and hearing loss did not appear until around 2020, which is later than other ferroptosis-related research fields.^[15,22]^ This delay may be related to the complex structure of the inner ear, the difficulty of establishing stable cochlear hair cell models, and the long-term focus on classical cell death pathways such as apoptosis and necrosis in previous auditory research.^[6,7,23]^

Since 2022, the number of publications on ferroptosis and hearing loss has increased rapidly, indicating growing interest in this emerging field. Previous studies have explored the role of ferroptosis pathways in various types of sensorineural hearing loss, including drug-induced ototoxicity, noise-induced hearing loss, age-related hearing loss, ischemia-reperfusion injury, and autoimmune-related auditory damage.^[6,8,24,25]^ In particular, studies by Mei et al and Hu et al in 2020 provided early evidence that pharmacological inhibition of ferroptosis with Fer-1 can reduce platinum-induced damage in HEI-OC1 cells and cochlear explant models.^[26,27]^ Subsequent studies using animal and cell models have further deepened the understanding of ferroptosis in auditory injury.^[28]^ However, current research is still mostly based on in vitro and ex vivo models. More studies using clinically relevant models are needed to clarify the role of ferroptosis in different types of hearing loss.^[15]^

### 4.2. Highly concentrated research output and insufficient international collaboration

China contributes the largest proportion of publications in this field, accounting for 85.3% of the total output. Research teams from institutions such as Fudan University and Shandong University are the main contributors. This rapid development may benefit from the solid foundation of otology research in China and the continuous support of national research programs.^[2,3]^ However, bibliometric analysis shows that contributions from European, American, and South Korean research groups are still relatively limited, and international collaborative publications are rare. Global cooperation in this field needs to be further strengthened.

In biomedical research, international and interdisciplinary cooperation is important for promoting the translation of basic discoveries into clinical applications. At present, studies on ferroptosis-related hearing loss are mainly based on cisplatin-induced ototoxicity models, while other types of hearing loss are less studied. Differences in experimental models, evaluation methods, and ferroptosis detection strategies may also affect the comparability of results between studies.^[6,29]^ Although current research is generally based on the ferroptosis concept proposed by Dixon et al,^[12]^ a unified international research network has not yet been formed. Future studies should promote broader cooperation between research centers and improve the standardization of experimental models and evaluation methods, which will help improve research reproducibility and promote clinical translation.^[14,30]^

### 4.3. Evolution of research hotspots: from general mechanisms to specific molecular targets

Keyword co-occurrence, clustering, and burst analysis together show a clear evolution of research interests in this field.^[15,22,31–33]^

The first stage (2020–2021) was the initial exploration period. Research mainly focused on broad biological processes related to ferroptosis, including iron accumulation, oxidative stress, inflammation, and regulated cell death pathways.^[5,7–9]^ Studies at this stage mainly aimed to confirm whether ferroptosis is involved in auditory injury, rather than exploring specific molecular regulators.

The second stage (2022–2024) saw increasing attention to molecular regulatory mechanisms. GPX4 became one of the core research hotspots with high burst intensity.^[31]^ As a key regulator of lipid peroxide metabolism, GPX4 plays an important role in maintaining cellular redox balance and has been widely studied in ferroptosis-related cochlear injury models.^[22,31]^ Meanwhile, ACSL4, a regulator related to ferroptosis sensitivity, has also received more attention and is often discussed together with GPX4 in ferroptosis regulation.^[32]^ This transition reflects the shift of the field from general descriptions of oxidative damage to in-depth studies of specific molecular pathways.

The third stage (2025–2026) shows an increasing focus on clinical relevance and disease applications. “Sensorineural hearing loss” has become a prominent emerging keyword, along with increasing attention to spiral ganglion neurons, stria vascularis, autophagy-associated ferroptosis, cisplatin-induced injury, and lipid peroxidation.^[20,22,33–35]^ Recent studies suggest that ferroptosis is involved in multiple components of the auditory system, including hair cells, spiral ganglion neurons, and cochlear supporting structures. This indicates that future research may further expand to disease-specific mechanisms, cell-type-specific regulation, and targeted protective strategies.

### 4.4. Imbalanced etiological distribution: exogenous injury models dominate, clinical disorders are understudied

Etiological distribution analysis shows that drug-induced hearing loss accounts for the largest proportion of included studies (46.67%), followed by noise-induced hearing loss (28.00%). In comparison, age-related (17.33%), hereditary (5.33%), and sudden sensorineural hearing loss (2.67%) are clearly underrepresented.^[6,7,24,36]^

Cisplatin-induced ototoxicity is the most commonly used experimental model, and there is relatively abundant evidence supporting its association with ferroptosis. Previous studies have shown that cisplatin affects iron metabolism and lipid peroxidation through multiple pathways: inhibiting system x₍c₎^−^ activity, altering FPN1 expression, regulating TFRC and DMT1, and activating NCOA4-mediated ferritinophagy.^[25,28]^ Due to its stable phenotype and good experimental reproducibility, cisplatin-induced ototoxicity remains a common model for studying ferroptosis in auditory injury.

Research on noise-induced hearing loss mainly focuses on oxidative stress regulation and antioxidant interventions, while ferroptosis-specific mechanisms need further investigation.^[7,8]^ Age-related hearing loss has been linked to age-related iron accumulation, decreased antioxidant capacity, mitochondrial dysfunction, and altered ferroptosis pathways.^[22]^ Sudden sensorineural hearing loss and hereditary hearing loss may also involve ferroptosis-related regulation, including changes in iron metabolism and antioxidant pathways, but related studies are very limited.^[36]^

The low proportion of studies on clinically common hearing disorders indicates a gap between current experimental research and clinical needs. Future studies should further explore ferroptosis-related mechanisms in more types of hearing loss using clinically relevant models.

### 4.5. Ferroptosis is part of a multi-pathway regulatory network

Current evidence suggests that ferroptosis does not occur as an isolated event in cochlear injury; it interacts with other biological processes including apoptosis, autophagy, and inflammatory responses.^[21,34,35]^

For example, NCOA4-mediated ferritinophagy promotes ferritin degradation and increases intracellular free iron levels, thereby enhancing ferroptosis sensitivity. Autophagy-related regulation has also been reported in cisplatin-induced auditory injury models.^[21]^ In addition, inflammatory mediators such as tumor necrosis factor alpha and interleukin-6 can affect ferroptosis processes by promoting redox imbalance and cellular stress.^[5,35]^ Several studies have observed the coexistence of apoptosis and ferroptosis in cisplatin-induced ototoxicity, suggesting that multiple regulated cell death pathways may jointly contribute to auditory injury.^[6,26]^

Therefore, future research should not only focus on individual cell death pathways but also explore the interactions between different regulatory networks. Clarifying the relationship between ferroptosis, apoptosis, autophagy, and inflammation will help develop more comprehensive hearing protection strategies.^[20,34,35]^

### 4.6. Core molecular pathways of ferroptosis-related hearing loss

Building upon the objective bibliometric findings, the following sections provide a contextualized discussion of the main mechanistic pathways identified in the core literature.

Based on current literature, ferroptosis-related cochlear injury is mainly discussed from 3 interconnected aspects: impaired antioxidant defense, dysregulated iron metabolism, and abnormal lipid peroxidation regulation.

First, impairment of the antioxidant defense system. The system x₍c₎^−^/glutathione/GPX4 pathway is recognized as one of the core regulatory pathways of ferroptosis. Decreased cystine uptake through system x₍c₎^−^ leads to reduced glutathione synthesis and impaired GPX4 activity, which causes accumulation of lipid peroxides and increases cellular susceptibility to ferroptosis.^[22,31]^

Second, dysregulation of iron metabolism. Changes in iron metabolism-related proteins lead to intracellular iron accumulation: increased expression of TFRC and DMT1, reduced FPN1-mediated iron export, and enhanced NCOA4-mediated ferritinophagy. Excess iron further promotes oxidative stress through Fenton reactions and increases ferroptosis sensitivity.^[25,28]^

Third, abnormal regulation of lipid peroxidation. ACSL4 is widely recognized as a key regulator of ferroptosis sensitivity. Increased ACSL4 activity promotes the incorporation of polyunsaturated fatty acids into membrane phospholipids, which increases the susceptibility to lipid peroxidation and promotes ferroptosis.^[32]^

These 3 pathways constitute the main molecular processes currently studied in ferroptosis-related auditory injury. Previous studies suggest that ferroptosis-related changes affect multiple cochlear cell types, including hair cells, stria vascularis cells, and spiral ganglion neurons, which may contribute to hearing loss.^[6,22,24]^ However, the specific role of ferroptosis in different cell types and different types of hearing loss still needs further study.

### 4.7. Ferroptosis-targeted strategies for hearing protection

Current research on ferroptosis-related protective strategies mainly focuses on 3 categories: small-molecule ferroptosis inhibitors, iron chelators, and natural compounds.^[26,27,30]^

Small-molecule inhibitors such as Fer-1, Liproxstatin-1 and 4-octyl itaconate have been reported to reduce lipid peroxidation or regulate antioxidant pathways (such as Nrf2 signaling) in cellular and animal models of auditory injury.^[26,27]^ Iron chelators such as deferoxamine can reduce iron-dependent oxidative damage by limiting free iron availability and inhibiting Fenton reaction-related processes.^[30]^

In recent years, natural compounds have attracted increasing attention as potential ferroptosis regulators. Compounds such as iridoid glycosides, hesperetin, paeoniflorin, maslinic acid, and timosaponin BII have been reported to regulate ferroptosis pathways through Nrf2/GPX4 signaling and NCOA4-mediated iron metabolism. Among them, timosaponin BII has shown protective effects in cisplatin-induced auditory injury models while maintaining the antitumor activity of cisplatin, which suggests its potential value for further research.

While ferroptosis-targeted compounds and iron chelators show promising protective effects in experimental models, current evidence is mostly based on in vitro cellular studies and in vivo rodent models. Translating these preclinical findings into clinical applications requires caution. Several critical challenges remain to be solved, including human pharmacokinetics, long-term safety, and the development of effective delivery systems to cross the blood-labyrinth barrier.

### 4.8. Limitations

Although this study provides a comprehensive overview of the research on ferroptosis in hearing loss, several limitations must be acknowledged.

First, regarding data collection, literature was exclusively retrieved from the WoSCC (SCI-E) database and restricted to English publications, which inevitably introduces single-database and language biases.^[37]^ Furthermore, the search strategy might be subject to potential keyword/search-term bias, and the data coverage for 2026 is incomplete since the retrieval was conducted in April 2026.

Second, methodological limitations exist in bibliometric software. The visualization network results are sensitive to software threshold settings. Additionally, while a rigorous manual normalization procedure was applied using a custom thesaurus, the inherent challenge of synonym fragmentation means that subtle subjective biases cannot be completely eliminated. The relatively small total sample size of the current literature and the inevitable citation lag effect may also obscure the most recent emerging trends.^[38]^

Third, concerning the content and interpretation of the findings, publications from China overwhelmingly dominate the current dataset (accounting for over 85% of total output), which may limit the global representativeness of the findings. The etiological classification applied in this study also involves a degree of subjectivity. Finally, and most importantly, while bibliometric analysis is a powerful observational tool for mapping knowledge structures, it is fundamentally incapable of establishing biological causality or demonstrating actual therapeutic effectiveness.^[39]^ Given that the current field is predominantly restricted to basic research, translating these bibliometric hotspots into clinical applications requires rigorous experimental and clinical validation.

## 5. Conclusion

In conclusion, this bibliometric and visualized analysis provides a comprehensive overview of the current international literature on ferroptosis in hearing loss from 2012 to March 2026 (partial year). The findings show that research interest in this field has increased rapidly in recent years, especially after 2022, reflecting growing attention to the role of ferroptosis in auditory disorders.

China has made the largest contribution in terms of publication output, but collaboration between institutions and countries is still limited. Keyword and co-citation analyses identify oxidative stress, cisplatin-induced ototoxicity, GPX4 regulation, iron metabolism, and lipid peroxidation as core research topics in this field.

Current studies mainly focus on the regulation of antioxidant systems, iron homeostasis, and lipid metabolism, and their relationship with cochlear injury. Therefore, targeting ferroptosis represents a promising experimental strategy for the prevention and treatment of hearing loss. However, further clinical trials, pharmacological optimizations, and breakthroughs in inner ear drug delivery are needed to translate these experimental findings into effective clinical therapies.

Overall, this study summarizes the development trends, research hotspots, and knowledge structure of ferroptosis-related hearing loss research. The findings can provide a useful reference for future basic and clinical studies on auditory injury and protective strategies.

## Author contributions

**Conceptualization:** Feng Qiu, Bin Ai.

**Data curation:** Feng Qiu, Ziyuan Chen.

**Formal analysis:** Feng Qiu.

**Funding acquisition:** Bin Ai.

**Investigation:** Feng Qiu.

**Methodology:** Feng Qiu.

**Project administration:** Bin Ai.

**Resources:** Huan Xie, Wusong Huang.

**Supervision:** Bin Ai.

**Validation:** Ziyuan Chen, Huan Xie, Wusong Huang.

**Visualization:** Feng Qiu, Ziyuan Chen.

**Writing – original draft:** Feng Qiu.

**Writing – review & editing:** Ziyuan Chen, Bin Ai.
